# Collectin-11 (CL-11) Is a Major Sentinel at Epithelial Surfaces and Key Pattern Recognition Molecule in Complement-Mediated Ischaemic Injury

**DOI:** 10.3389/fimmu.2018.02023

**Published:** 2018-09-06

**Authors:** Christopher L. Nauser, Mark C. Howard, Giorgia Fanelli, Conrad A. Farrar, Steven Sacks

**Affiliations:** MRC Centre for Transplantation, School of Immunology and Microbial Sciences, King's College London, Guy's and St. Thomas' NHS Foundation Trust, London, United Kingdom

**Keywords:** collectin-11, lectin pathway, complement system, innate immunity, renal ischaemia, renal transplantation

## Abstract

The complement system is a dynamic subset of the innate immune system, playing roles in host defense, clearance of immune complexes and cell debris, and priming the adaptive immune response. Over the last 40 years our understanding of the complement system has evolved from identifying its presence and recognizing its role in the blood to now focusing on understanding the role of local complement synthesis in health and disease. In particular, the local synthesis of complement was found to have an involvement in mediating ischaemic injury, including following transplantation. Recent work on elucidating the triggers of local complement synthesis and activation in renal tissue have led to the finding that Collectin-11 (CL-11) engages with L-fucose at the site of ischaemic stress, namely at the surface of the proximal tubular epithelial cells. What remains unknown is the precise structure of the damage-associated ligand that participates in CL-11 binding and subsequent complement activation. In this article, we will discuss our hypothesis regarding the role of CL-11 as an integral tissue-based pattern recognition molecule which we postulate has a significant contributory role in complement-mediated ischaemic injury.

## Introduction

**Figure 1:**
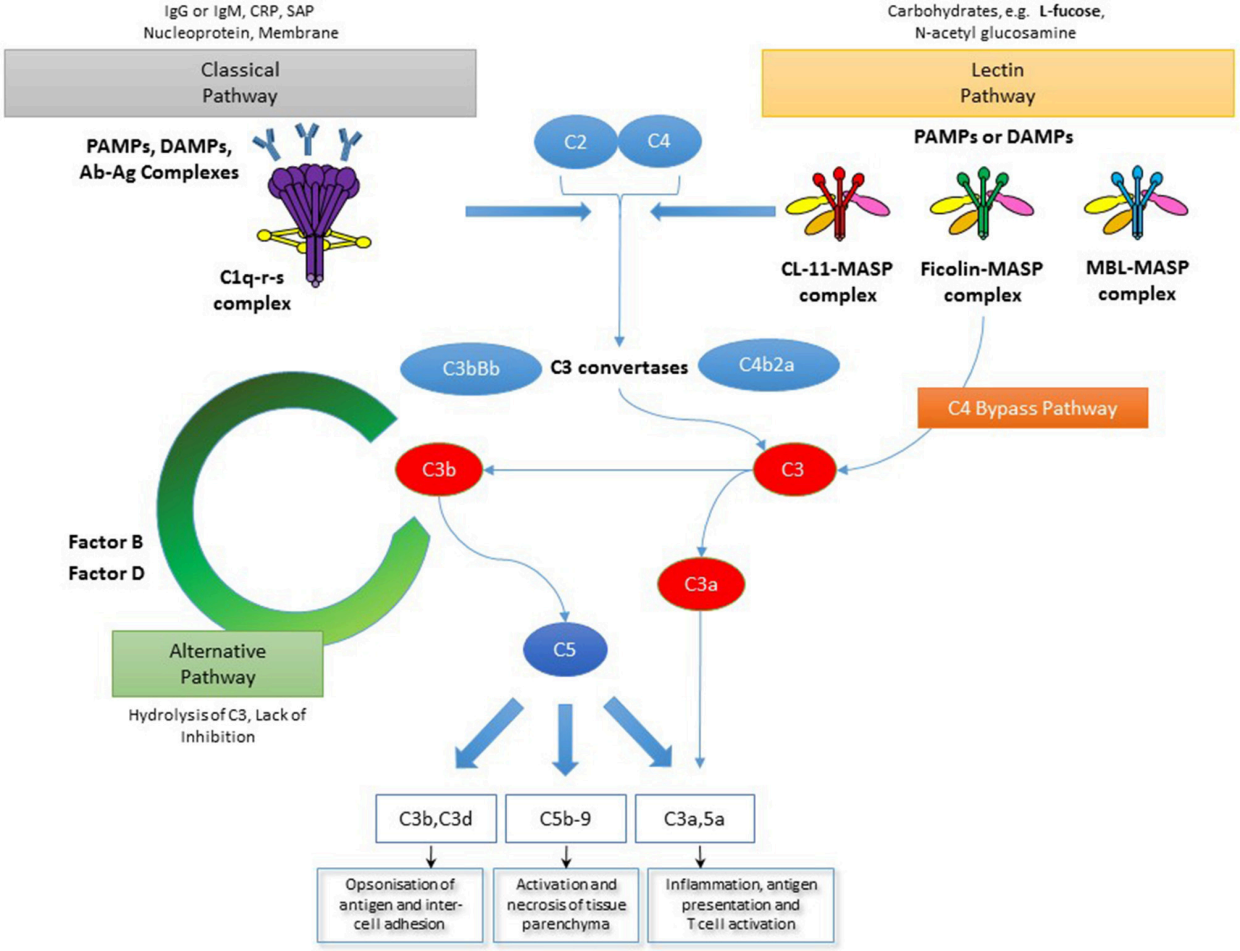
The complement system. The complement system is activated through three main pathways: classical, lectin and alternative. The classical and lectin pathways are activated by pattern recognition molecules binding to pathogen cell surfaces as well as infected and/or damaged cells. In the case of the classical pathway this manifests as C1q binding, most commonly via immunoglobulin. However, C1q can also bind other immune surveillance molecules or directly to disrupted structures via pathogen-associated molecular patterns (PAMPs) or damage-associated molecular patterns (DAMPs). Meanwhile, the lectin pathway is initiated by binding of collectins, such as MBL and CL-11, as well as ficolins, to PAMPs or DAMPs expressing carbohydrate ligands. Also shown in the diagram is a C4-bypass mechanism in which MASP-2 in association with lectin molecules directly cleaves C3. The alternative pathway is activated by C3b binding to cell surfaces and acts as an amplification process for the central complement component, C3, upon which both the lectin and classical pathways converge upon. Recently, MASP-1/3 was also shown to trigger the alternative pathway as well. Through a number of complement convertases the effectors of the complement system are generated. These are the anaphylatoxins, C3a and C5a, the membrane attack complex (MAC, C5b-9) as well as C3b and its metabolite C3d which mediate antigen opsonisation and cell-cell adhesion *(NB This is a generalized overview of the complement system as it specifically relates to the focus of this manuscript and is not meant to be a comprehensive depiction of all parts of the complement system)*.

We have seen over many years that innate immune defense systems mounted at epithelial surfaces perform multiple and often non-immune roles. The toll-like receptor system is a classic example that has transformed our perception about the origins of innate immunity and its roles in insect development (1, 2), antimicrobial defense (3) and in the pathogenesis of some inflammatory conditions (4–6). The complement system Figure 1 has been known about for longer (7), but the diversity of function and, in particular, its role at the interface between innate and adaptive immunity can now be re-visited as a typical model for innate immune function as well as a therapeutic target in a growing number of medical disorders.

## Early beginnings—local complement synthesis

For us, the story begins with early reports detailing the significance of local complement synthesis. Colten et al. authored a number of publications that highlighted the capacity of macrophages for producing a wide range of complement components (8, 9). Well cited papers on skin cells, neural cells, gut cells, cardiac cells, and others came into vision, showing that the principle of local synthesis was almost universal in resident parenchymal cells and migratory leukocytes (10–17). Our interest, as a nephrology group starting up in the late 1980s, was caught by kidney expression of a number of complement genes in the context of inflammatory renal disease (18, 19).

It became apparent that among the variety of intrarenal cells studied, the renal tubule cells were a prolific source of complement components. The renal tubule supports vital functions in maintaining health and blocking invasive pathogens such as uropathogenic *Escherichia coli*. The capacity of renal tubule cells for complement synthesis was first characterized through the work of Daha and others (20–22) in Leiden and further confirmed through histological examination of both healthy and diseased tissue by several research groups (23–25). The link between inflammatory conditions primarily affecting the renal tubules and a high degree of intrarenal complement expression was striking. Notably, these diseases included ischaemic injury of the newly transplanted kidney and transplant rejection (26–28).

At the time, the techniques of tissue- or cell-specific gene deletion were not well enough established to allow interrogation of local complement gene function, in the way that would be pursued now. However, we found that we could harness skills in mouse kidney transplantation to swap kidneys between wild-type and gene-deleted mice, in both directions, to illuminate the relative importance of local complement synthesis (29–31). Our initial focus was on C3, the central and most abundant component of the complement cascade in blood. We had already shown, in human kidney transplantation, that C3 has a significant contribution to the systemic complement pool through intrarenal synthesis (32).

## Complement and the transplanted kidney

The summation of the research including knockout mouse transplant studies and clinical observational studies has demonstrated a number of important principles. Namely, it showed that local synthesis of C3 had a disproportionate influence on ischaemia-reperfusion injury relative to the systemic pool (30), and an impact was also observed in the process of cell mediated rejection of MHC mismatched kidney transplants (31). It was evident from the analyses that the renal tubule cell was the primary target for complement deposition in these conditions, and the tubule cell was also the primary site of C3 expression (30).

The evidence additionally highlighted the role of donor antigen-presenting cells (APCs) as a source of complement (33). These cells, also known as donor passenger cells, reside in the interstitial space of the donor kidney, specifically around the renal tubules. Within the first 24 h after transplantation, these cells migrate into the recipient lymphoid system where they immunize the recipient against donor MHC antigen (34). Local production of complement was shown to modify the capacity of the APC to prime the antigen-specific T cells that mediate rejection (31, 35–37).

Considerable work has gone into identifying the complement effectors generated downstream of C3 that mobilize the inflammatory and adaptive immune functions against the transplant [reviewed in (38)]. These investigations have resulted in a deeper understanding both of the roles of the anaphylatoxins, C3a and C5a, (39) and the membrane attack complex (C5b-9) on immune cells and parenchymal tissue (40).

In addition to expressing core complement components and activating enzyme-precursors, tissue-resident and migratory cells also display receptors that detect a range of biologically active complement products formed downstream of C3 cleavage (41–43). This emphasizes the ability for cross talk between tissue-resident and migratory cells within the transplant setting. It is helpful to think of the different cells that produce and detect complement as nodes in a local network, whose functions bring together and amplify the innate immune response and regulate adaptive immunity.

## Local tissue defense

Presumably, the local synthesis of complement components serves to enhance the defense against invasive organisms. For example, the synthesis of C3 by renal tract epithelium potentially increases the efficiency with which locally invasive organisms are opsonised and subsequently eliminated at the point of entry. There is strong evidence for such a role, as the renal tubular epithelium constitutively expresses complement, and the production is rapidly upregulated in the presence of infection (44, 45). Further testament to this mechanism is that many common urinary pathogens have developed resistance to complement, including clinically relevant strains of gram negative pathogens (46–48). Not only have these strains been found to resist complement mediated lysis but they can also utilize complement to invade complement receptor expressing tubular epithelial cells (44, 49, 50). The C3b receptor, CD46, is one such receptor used by uropathogenic *E. coli* to evade extracellular defenses (45, 51) and is an illustration of the means by which complement resistant strains can gain an advantage against the host. Thus, the local pool of complement can be both a protector against infection and a source of tissue injury.

## Emergence of collectin-11

Whereas the last 20-years of research has taught us much about the effector functions of locally derived complement, our knowledge of the trigger mechanisms that localize tissue injury to a particular tissue compartment has lagged behind. This may be because the changes that induce complement activation are different for each organ and as such the studies in different organs have produced mixed and sometimes contradictory results [for more information refer to (52)]. Alternatively, it could be that the focus on circulating complement has not led us to the local mechanisms that drive complement-mediated disease. It is a common observation that measurement of circulating complement does not closely correlate with biopsy evidence of complement activation within an affected organ, and this may have delayed our understanding of local disease mechanisms. If only we understood more about the structures that triggered complement activation and how they are recognized in an organ such as the kidney, we would know more about how to detect and regulate harmful signals for health benefit.

We recently reviewed the evidence for the different pattern recognition molecules that could trigger complement activation in renal ischaemia-reperfusion injury and transplantation (52, 53). There, we considered whether the classical or lectin pathways could mediate the onset of ischaemia-reperfusion injury and found no conclusive evidence of a role for the classical pathway in the genesis of the renal injury within a murine model (54, 55). The lectin pathway also, at first, seemed not to have a key role in the induction of ischaemic renal injury, since the injury—at least in mice–was independent of C4, which is a component shared by both the classical and lectin pathways (56). However, we now believe that more recent findings on CL-11 and the coupled enzyme MBL associated serine protease-2 (MASP-2) reconcile these observations, both in the context of renal ischaemic epithelial cell damage and very possibly in retinal epithelial ischaemic damage (57–59).

CL-11 is a recently described member of the lectin family of pattern recognition molecules, with known antimicrobial functions and ability to trigger complement activation via the lectin pathway (60). Reported in 2006, CL-11 was at first named kidney collectin, or CL-K1, for its abundant expression in normal renal tissue (61, 62). The renaming of CL-11 is appropriate, since it is now known that the molecule is widely expressed (60). The most obvious expression site in the kidney is the renal tubule, for this structure encompasses the largest volume in the kidney, though CL-11 is also present in the glomerular mesangium and epithelium. Despite its strong presence in the kidney, the mean concentration of CL-11 in serum is just 284 ng/mL, by ELISA measurement. Furthermore, CL-11 is known to form heteromeric complexes with Collectin Liver 1 (CL-10) in the serum. Interestingly, this heterocomplex, CL-LK, has been shown to activate complement *in vitro* (63). However, we hypothesize that it is the local production of complement that accounts for tissue injury. Indeed, CL-11 has been shown to be produced by renal epithelial cells, whereas CL-10 has not been definitively shown to be expressed in the kidney (64) thereby making it less likely that the CL-LK heterocomplex is participating in renal injury.

CL-11 monomers have a similar structure to other C-type lectins such as MBL and consist of a globular head followed by a neck and a collagenous tail. The head contains a carbohydrate recognition domain (CRD), and the tail contains binding sites for MASPs which are required for complement activation. The CL-11 monomers form a triplet structure that self-combines to form oligomers with higher avidity of binding to ligand (65). Since hypoxia- or hypothermia-treated epithelial cells appear to bind CL-11 avidly (57–59), it is proposed that a change in presentation of the stress-induced cellular ligand for CL-11 underpins strong attachment of the oligomeric CL-11 complex. Whether this stress-induced pattern could involve a change in orientation or distribution of ligand, or increased expression or alteration of biochemical structure, is currently under investigation. However, clues to potential binding motifs or patterns can be gleaned from understanding the binding properties of other lectins. Mannose-Binding Lectin (MBL) is a well characterized C-type lectin similar to CL-11 in that it shares a similar structure with a CRD and collagenous tail, and binds oligosaccharide ligands in a calcium-dependent manner. In particular, MBL has a higher affinity for ligands that contain mannose or N-Acetyl-D-glucosamine residues (66). More recently, information has been gathered which further characterizes the MBL-oligosaccharide interaction, specifically in relation to MBL binding of lipopolysaccharide (67). Thus, our understanding of potential CL-11 ligands could be narrowed by considering and applying our knowledge of the glycan motifs that MBL recognizes.

Many molecules are normally glycosylated to some extent. In particular, fucosylated molecules are widely synthesized in normal tissues (68), and what is interesting to us is that L-fucose, the preferred monosaccharide recognized by CL-11, is also abundant in the proximal renal tubule (57). These are the very cells that express complement components in abundance. Therefore, the core components of the complement system including C3, C5 (23) and a lectin pathway trigger (CL-11) are expressed within the same hypoxia-sensitive segment of the renal tubule, where a potential binding ligand for CL-11 is also present. *In vivo* and *ex vivo* studies of epithelial cell injury suggest that hypoxia- or hypothermia-induced binding of CL-11 is followed by complement activation on the injured cell surface at sites that are specifically marked by CL-11. In a murine model of hypoxia-induced renal tubule cell stress, complement deposition was prevented by CL-11 deletion or by L-fucose blockade of the carbohydrate recognition domain of CL-11 (57). The protective effect of L-fucose blockade could also be demonstrated in wild type mice undergoing renal ischaemic insult (unpublished data). A similar injury mechanism also appears to occur in retinal pigmented epithelial cells, where hypoxia-induced membrane attack complex formation and CL-11 deposition correlate with sites of L-fucose expression (59). Thus, these findings may have potentially broad implications for diseases where complement mediated injury is thought to play a significant role.

## Closing the Gap

David and colleagues originally described the ability of renal tubule cells to spontaneously activate complement in the presence of normal human serum, and they attributed this to activation of the alternative complement pathway (69, 70). However, subsequent understanding of the lectin pathway and, in particular, the role of CL-11 now suggests another explanation (57). It points to a role for pattern recognition by CL-11 in contact with a damage-associated ligand on hypoxia-activated cells, and in turn the subsequent activation of the complement cascade by lectin-pathway associated serine proteases, i.e. MASPs. Although the alternative pathway may still play a role in hypoxia-mediated renal injury, the emerging data suggests that CL-11 is indispensable for triggering complement activation. Secondary activation of the alternative pathway could then occur either after the formation of C3b (which is an acceptor for factor B of the alternative pathway), or by MASP-1/3-mediated cleavage of complement factor D, which in turn cleaves factor B (60, 71). In the current model, CL-11 is expressed at physiological levels in the kidney and only substantially increases in binding to the tubule following cell stress. Complement activation then occurs at the sites where CL-11-MASP complexes form.

MASP-2 and its relationship with CL-11 are thought to play an essential role in this model. MASP-2 is one of the three MASPs that are physically linked with complement-activating lectins, including CL-11. MASP-2 differs from MASP-1 and MASP-3, in that only MASP-2 can directly cleave C3 in human and murine sera (64, 72). It is only recently that studies in gene-deleted mice have not only confirmed the importance of MASP-2 in mediating renal, cardiac and intestinal ischaemic injuries, but have shown that the injury in each case was C4-independent (30, 58, 73). The evidence supports a pathway of injury in which stress-induced ligand presentation leads to CL-11 binding and subsequent MASP-2-mediated triggering of complement activation using a route that involves the direct cleavage of C3 by MASP-2 (60, 71, 74). As indicated above, MASP-1/3-mediated activation of the alternative complement pathway could be a supplementary mechanism of injury triggered by CL-11.

**Figure 2:**
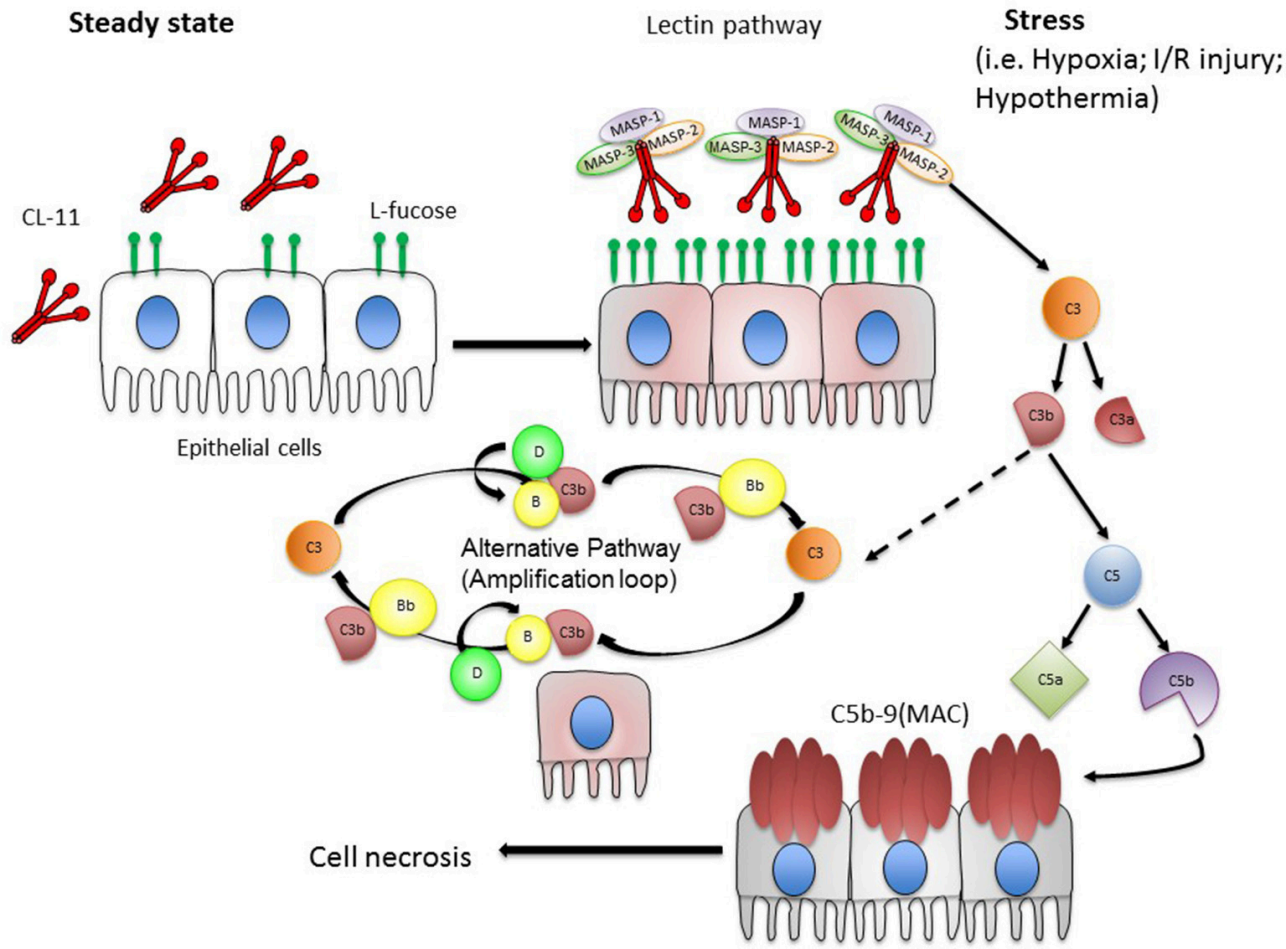
Hypothesis of local complement activation triggered by CL-11 on stressed epithelial cells. We hypothesize that under steady state conditions, CL-11 is produced and released from the basolateral surface and likely the luminal surface of epithelial cells (e.g., renal tubular epithelial cells and retinal pigmented epithelial cells). Upon stress, damaged epithelial cells display an abnormal pattern of L-fucose resulting in CL-11 binding to cell surface. CL-11/MBL-associated serine protease (MASP 1, 2 & 3) complexes become activated promoting downstream complement activation. In particular, MASP-2 is a key player at the site of tissue injury cleaving C3 in a C4-independent manner. During this process, the anaphylatoxins C3a and C5a are generated and C5 cleavage initiates the terminal pathway that culminates in the formation of the membrane attack complex (MAC). C3b formed by the lectin pathway can covalently bind to target cells and initiate the alternative pathway. C3b bound to factor B (C3bB) is cleaved by factor D to form the alternative pathway C3 convertase.

In total, the evidence suggests that the renal epithelial cell behaves as a unit of host defense and inflammation, in which CL-11 appears to integrate the detection of cell stress signals with activation of the complement system. We envisage that CL-11 is primarily a tissue-based pattern recognition molecule that binds damage associated molecular patterns (DAMPs) Figure 2. In the presence of tissue stress from non-infectious causes, the effector actions of CL-11 appear to be misdirected by glycan ligands that are inappropriately exposed on the renal tubule cell surface. Similar cellular responses to stress such as hypoxia probably exist at other surfaces, such as the retinal epithelium, where hypoxia-induced complement activation in the presence of CL-11 is also described (59). What remains unclear is the biochemical structure of the damage-related glycan ligands (and carrier molecules) recognized by CL-11. Future studies will need to elucidate the nature of the stress signal on cells and how it is induced by hypoxia to fully validate the hypothesis, and show whether the same or similar mechanism operates at other epithelial surfaces. As the identity of these DAMPs begin to emerge, it will be important to determine whether and how those exposed on renal tissue differ from the CL-11 ligands on other tissues and microbial structures, or indeed on developing tissues targeted by CL-11. There is little doubt that investigating CL-11 will provide new tools to prove the wider functions of immune surveillance at different sites, with the potential to develop new clinical agents for detecting or blocking specific patterns.

## Author contributions

CN and MH participated in manuscript writing, editing, and figure development. GF contributed to figure development. CF and SS contributed to manuscript editing, and writing, respectively.

### Conflict of interest statement

The authors declare that the research was conducted in the absence of any commercial or financial relationships that could be construed as a potential conflict of interest.
